# Population Abundance of Potentially Pathogenic Organisms in Intestinal Microbiome of Jungle Crow (*Corvus macrorhynchos*) Shown with 16S rRNA Gene-Based Microbial Community Analysis

**DOI:** 10.1155/2013/438956

**Published:** 2013-08-24

**Authors:** Isamu Maeda, Mohammad Shohel Rana Siddiki, Tsutomu Nozawa-Takeda, Naoki Tsukahara, Yuri Tani, Taki Naito, Shoei Sugita

**Affiliations:** Faculty of Agriculture, Utsunomiya University, 350 Minemachi, Utsunomiya 321-8505, Japan

## Abstract

Jungle Crows (*Corvus macrorhynchos*) prefer human habitats because of their versatility in feeding accompanied with human food consumption. Therefore, it is important from a public health viewpoint to characterize their intestinal microbiota. However, no studies have been involved in molecular characterization of the microbiota based on huge and reliable number of data acquisition. In this study, 16S rRNA gene-based microbial community analysis coupled with the next-generation DNA sequencing techniques was applied to the taxonomic classification of intestinal microbiome for three jungle crows. Clustering of the reads into 130 operational taxonomic units showed that at least 70% of analyzed sequences for each crow were highly homologous to *Eimeria* sp., which belongs to the protozoan phylum *Apicomplexa*. The microbiotas of three crows also contained potentially pathogenic bacteria with significant percentages, such as the genera *Campylobacter* and *Brachyspira*. Thus, the profiling of a large number of 16S rRNA gene sequences in crow intestinal microbiomes revealed the high-frequency existence or vestige of potentially pathogenic microorganisms.

## 1. Introduction

The Jungle Crows (*Corvus macrorhynchos*) are very adaptable to human habitats and are able to survive on a wide range of food sources. They are extremely versatile in their feeding and prefer environments with an abundant supply of large garbage or carcasses in cities and coastal areas [1]. Microorganisms' populations that collectively comprise microbiota in crow's digestive system are associated with rearing conditions and environment. The intestinal microbial communities do not only have an important role in host metabolism [2] but also relate with zoonosis and food poisoning to human, animals, and birds [3]. Crows are considered as potential sources for fecal contamination of water supplies and foods, and the pathogens are transmitted through the fecal-oral route when humans would consume those contaminated foods or water [4]. However, information on microbiotas of crow intestine, which might harbor pathogenic organisms to humans and livestock, remains to be insufficient or obscure. Therefore, it is important for public health to characterize the intestinal pathogens within the gut of Jungle Crow, and more efforts should be given for understanding their profiles and transmission of diseases to humans and animals.

Gut microorganisms have been isolated and characterized by a variety of methods such as culture-based assays, culture-independent DNA sequencing approaches using the conventional Sanger sequencing, and the fluorescence *in situ *hybridization targeting the 16S  rRNA gene [5]. Most of such traditional approaches are limited in scope. Culture-based assays bias bacterial profiling towards culturable fractions. DNA-based traditional approaches have targeted to phylogenetic genes, which provided limited information on the public health relevance, especially when a large amount of microbial groups must be classified [5]. That weakness becomes obvious in conventional Sanger's sequencing, which has limitation in terms of amount of reads produced and affordability.

In recent years, metagenomics using the next-generation sequencing has been chosen in analyzing complex microbial communities and functions. The next-generation sequencers are capable of massively parallel sequencing of thousands or millions of amplified DNA molecules in a single run and do not require the conventional cloning and amplification in bacteria when compared to the traditional Sanger capillary sequencers [6]. These technologies have enabled metagenomics to become widespread, routine, and inexpensive, rather than requiring significant production-scale efforts [5, 7]. Metagenomic study offers a powerful lens for viewing the comprehensive and unbiased assessment of microbial diversity within the complex gut ecosystem by allowing examination of organisms not easily cultured in laboratory [8, 9].

Although microorganisms in the digestive tract of Jungle Crow have been studied by using conventional culture-based assays [3], sufficient coverage of nonabundant and unculturable microbial groups in the gut have not been obtained. Interestingly, in spite of its importance in the public health relevance, no information on crow gut microbiota is available. The present study aims to characterize the microbial community within the intestine of Jungle Crow based on information of thousands of reads targeted to microbial 16S  rRNA genes obtained by the next-generation DNA sequencing.

## 2. Materials and Methods

### 2.1. Preparation of Crow Intestinal Contents

All procedures involving crows were performed in accordance with the guidelines of Utsunomiya University (animal experiment permission A11-0012). Adult three Jungle Crows (*Corvus macrorhynchos*) were caught by traps set at the Experimental Farm of Utsunomiya University located in Moka city during their nonbreeding season (October 2011, December 2011, and January 2012). Permits to trap crows were obtained from the Tochigi prefecture (number 0010). All the materials that came in contact at processing and preparation of samples were cleaned with disinfectant or sterilized. The crows were euthanized by an overdose of pentobarbital sodium (50 mg/kg body weight). The crows were killed using sharp knife and allowed to drain all blood. Then, the crows were dissected immediately with scissors. The intestine was occluded by the occluding clamps, aseptically removed from the abdominal cavity of crow, and soaked in distilled water in a Petri dish. All the contents were gently squeezed out of the intestines to the distilled water and transferred into polypropylene centrifuge tubes. The intestinal contents were harvested with centrifugation for 5 min at 4°C with 10,000 rpm.

### 2.2. DNA Extraction and Purification from Intestinal Content

Genomic DNA was isolated and purified from fresh intestinal content by using QIAamp DNA Stool Mini kit (Qiagen, Tokyo, Japan). For this purpose, 200 *μ*g fresh weight of sediment of crow intestinal content was thoroughly homogenized according to the manufacturer's protocol. Finally, genomic DNA was dissolved with 200 *μ*L buffer AE (10 mM Tris-Cl, 0.5 mM EDTA, pH 9.0). DNA purity and concentration were analyzed spectrophotometrically using the SmartSpec plus (BioRad, Tokyo, Japan). The extracted 10−17 *μ*g DNA was stored at −20°C until use.

### 2.3. PCR Conditions

Twenty-five to fifty nanograms of isolated DNA were transferred to a PCR tube containing 50 *μ*L of a polymerase chain reaction (PCR) mixture containing LA *Taq* DNA polymerase (TaKaRa Bio, Shiga, Japan). For DNA evaluation, the eubacterial 16S  rRNA gene sequence intervened between UNIV341F (5′-CCTACGGGAGGCAGCAG-3′) and UNIV907R (5′-CCCCGTCAATTCCTTTGAGTTT-3′) was amplified. For pyrosequencing, the eubacterial 16S rRNA gene sequence intervened between the 27F (5′-AGAGTTTGATCMTGGCTCAG-3′) and 519R (5′-GWATTACCGCGGCKGCTG-3′) sites was amplified [10]. MA-27F, to which multiplex identifiers (MIDs), MID-151 (5′-CCATCTCATCCCTGCGTGTCTCCGACTCAGTGCTAGTCAG-3′), MID-152 (5′-CCATCTCATCCCTGCGTGTCTCCGACTCAGTGTATCACAG-3′), and MID-153 (5′-CCATCTCATCCCTGCGTGTCTCCGACTCAGTGTGCGCGTG-3′), were attached, and MB-519R (5′-CCTATCCCCTGTGTGCCTTGGCAGTCTCAGGWATTACCGCGGCKGCTG-3′) were provided from Hokkaido System Science (Sapporo, Hokkaido, Japan). MIDs distinguished the amplified DNA fragments derived from three Jungle Crows. The thermal cycling conditions were composed of a denaturing step at 94°C for 1 min, 30 thermal cycles of 94°C for 30 s, 60°C for 30 s, and 68°C for 1 min, and an additional extension step at 72°C for 5 min. The PCR products were analyzed by the agarose (1.2% w/v) gel electrophoresis in TBE buffer (89 mM Tris-borate pH 8.3, 2 mM EDTA). The PCR fragments were stained with ethidium bromide and visualized with a UV transilluminator after electrophoresis. The PCR products for pyrosequencing were purified using QIAquick PCR purification kit (Qiagen, Tokyo, Japan).

### 2.4. Pyrosequencing and Population Analysis

The PCR products with MIDs, whose quality was analyzed with a bioanalyzer (Agilent 2100, Agilent Technologies, Palo Alto, CA, USA), were equally mixed and subjected to commercially available pyrosequencing using the Roche Genome Sequencer GS FLX+ system (Hokkaido System Science, Sapporo, Hokkaido, Japan). Determined nucleotide sequences not containing unspecified nucleotide and with read length of longer than 250 bp were extracted as passed-filter (PF) reads and categorized into 3 groups according to MIDs, subjected to the BLAST search against the DNA Data Bank of Japan (DDBJ) bacterial 16S  rRNA gene database, and converted to the data sets composed of taxonomy ID and genus, based on information of a top hit where alignment length was more than 30% of submitted nucleotide sequence. Then, genera showing the BitScores higher than 680 were chosen for further analysis. Finally, the genera identified were confirmed by the Ribosomal Database Project (RDP) [11] Classifier with the confidence threshold of 80%.

### 2.5. Phylogenetics and Statistics

Using RDP, key tag and primer sequences were trimmed off and sequences with low quality were removed from the PF reads, and then the qualified sequences were aligned and compared by RDP to create column formatted distance matrix data. Finally, operational taxonomic units (OTUs) were created based on the distance matrix data by mothur [12]. By counting the number of sequencing in each OTU, the reciprocal of the Simpson index was calculated as *α* diversity, which represents observed richness within a group, and the *θ*_YC_ distance of Yue and Clayton was calculated as *β* diversity, which represents compositional heterogeneity between two habitats [13]. A heatmap was created based on the OTU data by R 3.0.1 for Windows.

## 3. Results and Discussion

### 3.1. Evaluation of Extracted DNA and Pyrosequencing from Crow Intestinal Contents

**Figure 1:**
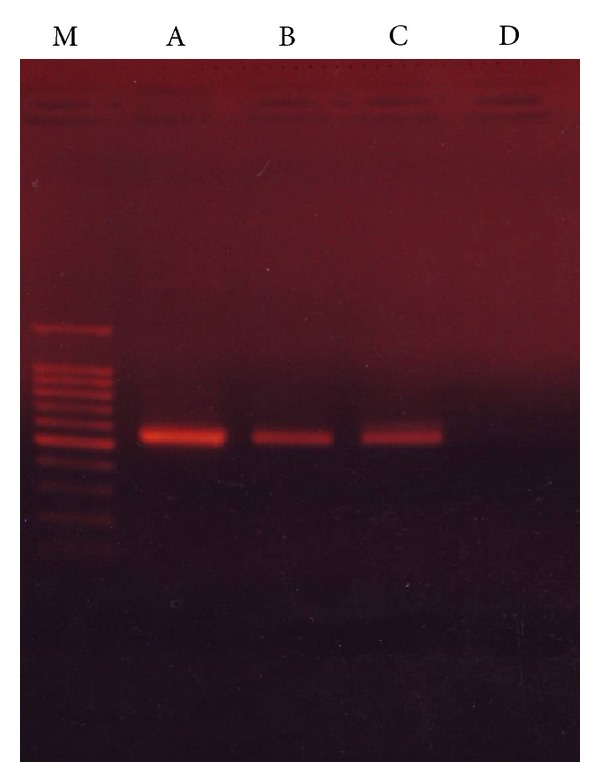
Amplified partial fragment of 16S  rRNA gene from crow 1 (A), crow 2 (B), and crow 3 (C) for evaluation of intestinal microbiomes. Control PCR was performed under the same conditions except for without template DNA (D). Molecular size marker (M) shows 1.5, 1.0, 0.9, 0.8, 0.7, 0.6, 0.5, 0.4, 0.3, 0.2, and 0.1 kb from top to bottom.

For analyzing the quality of total DNA extracted from crow intestinal microbiota, PCR was attempted using LA *Taq *DNA polymerase in combination with UNIV341F and UNIV907R primers. The expected products size of 550 bp with sufficient DNA concentrations was obtained from intestinal DNA extracted from three specimens of crow intestinal content (Figure 1). Without template DNA for PCR, DNA fragments were not detected, indicating that the amplified DNA fragments were derived from intestinal microbial DNA.

For pyrosequencing of 16S  rRNA genes in crow intestinal microbiome, PCR was attempted using LA *Taq* DNA polymerase in combination with MA-27F and MB-519R primers. The amplified DNA fragments had an almost uniform size distribution between 480 bp and 490 bp. As a result of pyrosequencing, more than 10,000 PF reads were obtained for each crow (Table 1). A total of 303 reads for crow 1, 4,563 reads for crow 2, and 3,143 reads for crow 3 matched the DDBJ bacterial 16S  rRNA gene sequences with the confident level.

### 3.2. Microbial Community Analysis

**Figure 2:**
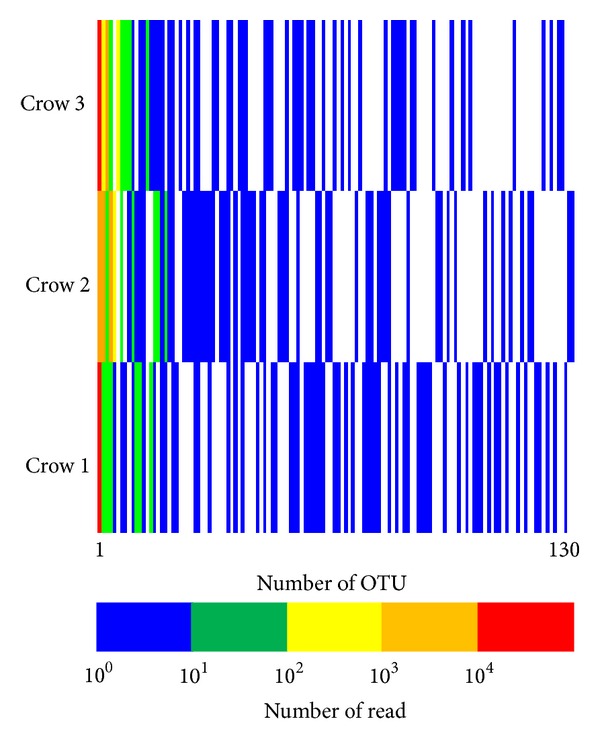
Heatmap representation of the clustered distant matrix data based on the pyrosequencing for 16S  rRNA genes of crow intestinal microbiome. Number of OTU: numbers of operational taxonomic unit; number of read: numbers of read clustered.

Using RDP, tag and primer sequences were removed from the PF reads, and short sequences that did not contain the 519R sequence were excluded. A total of 41,355 reads from the three crows were analyzed, of which 130  OTUs were clustered at the distance label of 0.05 (Figure 2). OTU1 occupied 98% of the analyzed reads obtained from crow 1, 70% of those from crow 2, and 81% of those from crow 3. Among the analyzed reads, 97% and 96% of the total numbers were clustered into OTU1, OTU2, and OTU4 in crow 2 and OTU1 and OTU3 in crow 3. The sequences clustered within OTU1 were highly homologous to those of 16S  rRNA genes in the protozoan phylum *Apicomplexa*. The highest identity (96% in 469 bp, 1% gap) was obtained with that of *Eimeria praecox*.

**Figure 3:**
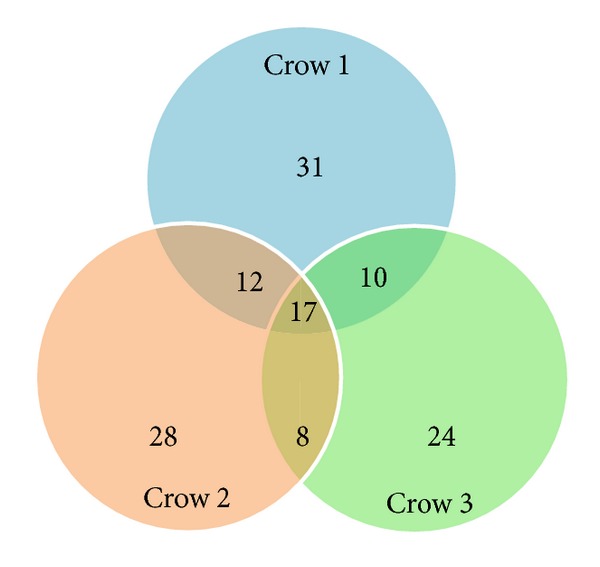
Venn diagram showing numbers of shared and unique taxa among the three crows.

Alpha and beta diversities were dependent on bacterial abundance (Table 1), which was shown as the numbers of read within OTU2-130. Sixteen OTUs for bacteria and one OTU (OTU1) for *Eimeria* sp. were shared among the three crows (Figure 3). However, uniquely found OTUs in each crow were composed of small numbers of reads (<10), except for OTU6 (126 reads) and OTU14 (22 reads) in crow 3.

### 3.3. Taxonomy of Potentially Pathogenic Bacteria

Among the 8009  PF reads in the three crows matched with sequences in the bacterial 16S  rRNA gene databases, the sequences mostly clustered into OTU2 were abundantly found (12% in crow 1, 54% in crow 2, and 4.0% in crow 3) and identified as the gene of the genus *Helicobacter*. The highest hits were obtained against *H. suncus* (99% identity) and *H. pametensis* (99% identity). Within the crow 1 and crow 3 intestinal microbiotas, *Lactococcus* sp. and *Campylobacter* sp. (OTU3) were the most dominant bacteria (16% in crow 1 and 76% in crow 3), respectively. The highest hit among the genus *Campylobacter* was obtained against *C. jejuni* (100% identity). Among the PF reads obtained with the crow 2 microbiota, the sequences mostly clustered into OTU4 were secondly abundant (28%) and showed 100% identity with the gene of *Brachyspira pilosicoli*.

### 3.4. Possibility of the Jungle Crow as a Vector of Pathogen

Although the genus *Helicobacter* consists of over 20 recognized species,* H. pylori*, whose infection represents a key factor in the etiology of various gastrointestinal diseases [14], was not found in the three crows. The gene sequences of *H. suncus* and *H. pametensis*, which were isolated from the house musk shrews [15], wild birds, and a domestic swine [16], were commonly found among the intestinal microbiotas of three crows. However, it has not been well investigated whether these bacteria possess pathogenicity as observed in *H. pylori*. The fact that the sequences highly homologous to the gene of *B. pilosicoli*, which is a zoonotic intestinal spirochaete and causes avian intestinal spirochaetosis [17], and to the gene of foodborne pathogen *Campylobacter jejuni* [18] exist in the crow intestinal microbiotas suggests that the crow's feces is a potential source that spreads bacterial infectious diseases harmful for domestic fowls and humans. 

This is the first study in which a next-generation sequencing approach to characterize the crow intestinal microbiotas was carried out. Based on bioinformatics, which can handle a huge amount of data coupled with the next-generation sequencing of PCR-amplified DNA, the existence and vestige of pathogenic organisms could be clarified. Jungle Crows do not only acquire pathogenic microorganisms from their native habitats, but also return them via excretion. Garbage is preferred by Jungle Crows (*Corvus macrorhynchos*) than Carrion Crows (*Corvus corone*) [1]. These crows are highly adaptable in urban area, and their droppings are everywhere; thus contamination is inevitable. The discovery by this study demonstrating inhabitation of large population of *Eimeria* sp., whose pathogenesis to chickens has been shown [19], in crow intestine also supports the hypothesis that Jungle Crows might be potential vectors and natural reservoirs of the causal agents of some diseases and virulent strains to domestic fowls and humans. 

## 4. Conclusions

Herein, the crow intestinal microbiotas were characterized in terms of bacterial and protozoan genera that potentially cause some diseases to domestic fowls and in some cases to humans using pyrosequencing of 16S  rRNA genes. Diversity of microbial populations by each crow clarified that *Eimeria* sp. in the protozoan phylum *Apicomplexa* was highly abundant in all three microbiotas of crow intestine and bacterial genera such as *Campylobacter* and *Brachyspira* were dominantly included depending on the individual crow bodies. The obtained information suggests roles and importance of Jungle Crows on transferring pathogenic microorganisms within the human habitat.

## Figures and Tables

**Table 1:** Summary of numbers of reads, numbers of OTU, and diversity measurements.

Crow	Sampling month, year	Number of PF read	Number of analyzed read	Distance label	Number of OTU	*α* diversity	*β* diversity
1	October, 2011	17635	15050	0.05	70	1.03	Crows 1-2, 0.270; Crows 1–3, 0.087; Crows 2-3, 0.165
2	December, 2011	14757	12142	0.05	65	1.90	
3	January, 2012	17712	14163	0.05	59	1.48	
